# Rootstock and Crop Load Effects on ‘Honeycrisp’ Photosynthetic Performance and Carbohydrate Accumulation

**DOI:** 10.3390/plants12234035

**Published:** 2023-11-30

**Authors:** Claudia Baldassi, Anna Berim, Stefan Roeder, Pasquale Losciale, Sara Serra, David R. Gang, Stefano Musacchi

**Affiliations:** 1 Tree Fruit Research and Extension Center, Washington State University, Wenatchee, WA 98801, USA; claudia.baldassi@wsu.edu (C.B.); stefan.roeder@wsu.edu (S.R.); sara.serra@wsu.edu (S.S.); 2 Department of Horticulture, Washington State University, Pullman, WA 99164, USA; 3 Institute of Biological Chemistry, Washington State University, Pullman, WA 99164, USA; aberim@wsu.edu (A.B.); gangd@wsu.edu (D.R.G.); 4 Department of Soil, Plant and Food Sciences, University of Bari “Aldo Moro”, 70126 Bari, Italy; pasquale.losciale@uniba.it

**Keywords:** *Malus* × *domestica* Borkh., gas exchange, chlorophyll fluorescence, sorbitol, starch

## Abstract

Rootstock selection and crop load adjustment are key practices in apple orchard management; nevertheless, the effects of rootstocks and crop load levels on important physiological processes of the scions, such as photosynthetic performance and carbohydrate accumulation, are still unclear. To investigate the impact of different rootstocks and crop load levels on scion photosynthesis and carbohydrate buildup, in 2020, ‘Honeycrisp’ trees grafted on rootstocks ‘G.41’, ‘G.935’, and ‘M.9-T337’ were thinned to low and high crop load levels, and photosynthetic performance and carbohydrate accumulation in leaves and fruit were evaluated. Leaves from ‘G.935’ showed the highest net photosynthesis and electron use efficiency of photosynthesis and the lowest activity for non-net carboxylative processes, all together indicative of enhanced photosynthetic performance. High crop load determined an increase in gas exchange, suggesting a positive feedback of high fruit competition on carbon assimilation. While rootstock ‘M.9-T337’ showed a higher accumulation of starch in leaves, no pattern regarding the composition of leaf-soluble sugars among rootstocks could be identified. Conversely, by the end of the harvest season, leaves from low-cropping trees had higher fructose, glucose, and sorbitol than those from high-cropping trees, but differences in starch content were not significant. Fructose and sorbitol concentrations were affected by rootstock and crop load, respectively. Overall, this study showed that high cropping enhanced photosynthesis in ‘Honeycrisp’ apple and determined lower accumulation of some soluble carbohydrates (fructose, glucose, sorbitol) in leaves. This study also provided insights into how rootstocks affect photosynthetic performance of ‘Honeycrisp’, highlighting ‘G.935’ as the rootstock conferring the highest photosynthetic capacity under the present experimental conditions.

## 1. Introduction

Washington State is the top apple producer in the USA and the second worldwide, behind China [1,2]. Similar to other fruit-growing regions in the world, the apple-growing area of Washington State is characterized by a semiarid climate [3], with intense solar radiation and hot temperatures in the summer [4]. Day temperatures exceeding 30 °C are commonly recorded during the apple growing season [4], and climate change will likely increase the occurrence and intensity of heatwaves [5,6]. Under these environmental conditions, critical physiological and metabolic processes of apple trees are challenged [7,8].

Photosynthesis, considered one of the most heat-sensitive physiological processes [9,10], supplies plants with energy in the form of carbohydrates to support plant metabolism. Carbohydrates not only sustain apple trees’ growth and development [11] but also are key compounds for fruit quality, as they contribute to flavor [12,13], texture [14], and dry matter content [15]. The principal carbohydrates found in apple fruits are starch, fructose, glucose, sorbitol, and xylose [13]. Sorbitol, a sugar alcohol, is the primary phloem-translocated photosynthate in apple [12], and once inside the fruits, it is mainly converted into fructose and stored in vacuoles [16].

Rootstock selection and optimal crop load level are two of the most important choices in establishing and managing commercial apple orchards. While crop load plays a key role in controlling biennial bearing, rootstocks are essential for apple propagation and tree vigor control. Some of the most adopted rootstocks in modern orchards are ‘M.9-T337’ (dwarfing), a clone of the popular ‘M.9’ rootstock, ‘G.41’ (dwarfing), and ‘G.935’ (semi-dwarfing), both from the Geneva^®^ rootstock series of New York [17]. The effects of both rootstock and crop load on apple tree photosynthesis and carbohydrate accumulation have been the object of extensive research over time [18,19]. However, to date, results are contradictory, and it is still not clear how rootstock’s dwarfing capacity and crop load level affect the photosynthetic performance and carbohydrate accumulation of apple scions [20,21]. Photosynthetic performance has been documented to either decline [22] or increase [23,24,25,26] with increasing rootstock vigor. It has been observed that dwarfing rootstocks induce starch accumulation while depleting soluble sugars [23,27]. However, a case of increased leaf-soluble sugar concentration induced by super dwarfing rootstock (P.22) adjusted at 150 inflorescences·tree^−1^ has also been described [28]. Regarding crop load, increased fruit level on tree in different apple cultivars was associated with either enhancement [21,29,30] or decline of photosynthetic efficiency [31], and with either overaccumulation [31] or reduction in leaf carbohydrates [21,29,30]. It is clear that specific scion–rootstock interactions lead to these differences in results obtained by various researchers.

This study aimed to investigate the effects of rootstocks with different vigor levels (the Geneva^®^ rootstocks ‘G.41’ and ‘G.935’, and the commercial standard ‘M.9-T337’) and effects of varying cropping levels on ‘Honeycrisp’ photosynthetic performance and carbohydrate accumulation. In addition to providing further evidence on the role of rootstock and crop load in the regulation of apple tree photosynthesis and carbohydrate metabolism, the choice of using rootstocks from the Geneva^®^ series in this research provided the opportunity to investigate the performance of two of these rootstocks under Washington State growing conditions.

## 2. Results

### 2.1. Photosynthesis and Chlorophyll Content

Rootstock significantly affected net photosynthesis (Pn) and net photosynthesis expressed as electron transport rate (J_CO_2__) 98 days after full bloom (DAFB, *p* = 0.005), 115 DAFB (*p* < 0.001), and 129 DAFB (*p* < 0.001) (Table 1). ‘G.935’ consistently induced the highest Pn and J_CO_2__ (Table 1). Crop load too had a significant effect on Pn and J_CO_2__ 98 DAFB (*p* = 0.041), 115 DAFB (*p* < 0.001), and 129 DAFB (*p* < 0.001) (Table 1). Additionally, crop load had a significant effect on Pn and J_CO_2__ 162 DAFB, after fruit harvest (*p* = 0.036, Table 1). Beginning at 98 DAFB, low crop load induced, on average, lower Pn and J_CO_2__ values at all time points (Table 1).

Intercellular CO_2_ concentration (Ci) was significantly different among rootstocks at all time points, except at 65 DAFB and 98 DAFB (Table 1). ‘G.935’ recorded the lowest Ci, except at 83 DAFB, when ‘G.41’ showed the lowest value (238 μmol mol^−1^, Table 1). Crop load affected Ci at 83 DAFB (*p* = 0.002) and at 129 DAFB (*p* = 0.002), and high crop load recorded the highest values (282 μmol mol^−1^ and 325 μmol mol^−1^, respectively, Table 1). Transpiration rate (E) was affected by rootstocks at 162 DAFB only (*p* = 0.015). ‘G.935’ had the lowest E (0.99 mmol m^−2^ s^−1^) and ‘M.9−T337’ had the highest (1.88 mmol m^−2^ s^−1^). Crop load affected E at 83 DAFB (*p* = 0.032), 115 DAFB (*p* = 0.001) and 129 DAFB (*p* < 0.001). In general, E increased with increasing cropping level (Table 1). Similar to E, stomatal conductance (gs) was affected by rootstocks only at 162 DAFB (*p* = 0.013), with ‘G.935’ inducing the lowest value on average (0.056 mol m^−2^ s^−1^) and ‘M.9−T337’ the highest (0.121 mol m^−2^ s^−1^).

The effect of crop load on gs paralleled that on E and significant differences were observed 115 DAFB (*p* = 0.004) and 129 DAFB (*p* < 0.001), with the highest cropping level inducing the highest gs (Table 1). Electron use efficiency of photosynthesis (efCO_2_) was affected by rootstock at most time points, while crop load only had a significant impact at 162 DAFB (*p* = 0.003, Table 1). In general, rootstock ‘G.935’ showed the highest efCO_2_ values across measurements and the high crop load recorded the highest efCO_2_ at 162 DAFB (0.46). The parameter maximum efficiency of photosystem II in the light (*Fv’*/*Fm’*) was not affected by either rootstock or crop load at any time point (Table 1). Effective quantum yield of photosystem II (Φ_PSII_) and electron transport rate (ETR) showed the same pattern, with both being affected by crop load at 115 DAFB (Φ_PSII_ *p* = 0.016, ETR *p* = 0.019) and at 129 DAFB (Φ_PSII_ *p* = 0.003, ETR *p* = 0.004). For both variables, the high crop load treatment recorded the highest average values (Table 1). Concerning the residual absorbed energy used for non-carboxylative processes (J_NC_), crop load effect was significant only at 65 DAFB (*p* = 0.001) and rootstock significantly affected this parameter only at 83 DAFB (*p* = 0.002). Between crop load treatments, the high crop load scored the highest value on average (119.8 μmol m^−2^ s^−1^), and among rootstocks ‘M.9-T337’ recorded the highest value (113.5 μmol m^−2^ s^−1^), while ‘G.41’ the lowest (85.3 μmol m^−2^ s^−1^) (Table 1). Finally, the electron use efficiency of non-carboxylative processes (efNC) was affected by rootstock at every time point, except for 65 DAFB and 162 DAFB (Table 1). ‘G.935’ recorded the lowest efNC, with the only exception of 83 DAFB, when ‘G.41’ had the lowest value (0.601, Table 1). The effect of crop load on efNC was significant only at 162 DAFB (*p* = 0.002), and the highest value was observed with low-cropping trees (0.539).

Rootstock effect on leaf chlorophyll content (SPAD) was significant only at 65 DAFB, with ‘G.935’ leaves reporting the highest SPAD values on average (49.5) (Table 1). Crop load did not affect leaf chlorophyll content at any time point.

Interactions between rootstock and crop load were found significant only at 115 DAFB for the following parameters: Pn, E, J_CO_2__, efCO_2_, and efNC (Table 1 and Table 2). Pn and J_CO_2__ showed the same pattern, with ‘G.935’, high crop load recording the highest values (Pn 18.5 μmol m^−2^ s^−1^, J_CO_2__ 73.8 μmol m^−2^ s^−1^) and ‘G.41’, low crop load recording the lowest (Pn 6.4 μmol m^−2^ s^−1^, J_CO_2__ 25.7 μmol m^−2^ s^−1^). The combination ‘G.935’, high crop load, also had the highest E (4.76 mmol m^−2^ s^−1^), while ‘G.41’ and ‘G.935’ thinned to low crop load recorded the lowest values, 0.93 and 1.31 mmol m^−2^ s^−1,^ respectively. With efCO_2_, the lowest value was found for ‘M.9-T337’, high crop load (0.273), and the highest for ‘G.935’, low crop load (0.438). Finally, ‘M.9-T337’ thinned to high crop load showed the highest efNC (0.727), while ‘G.935’, also thinned to high crop load, had the lowest efNC (0.562).

### 2.2. Leaf Non-Structural Carbohydrates

**Figure 1:**
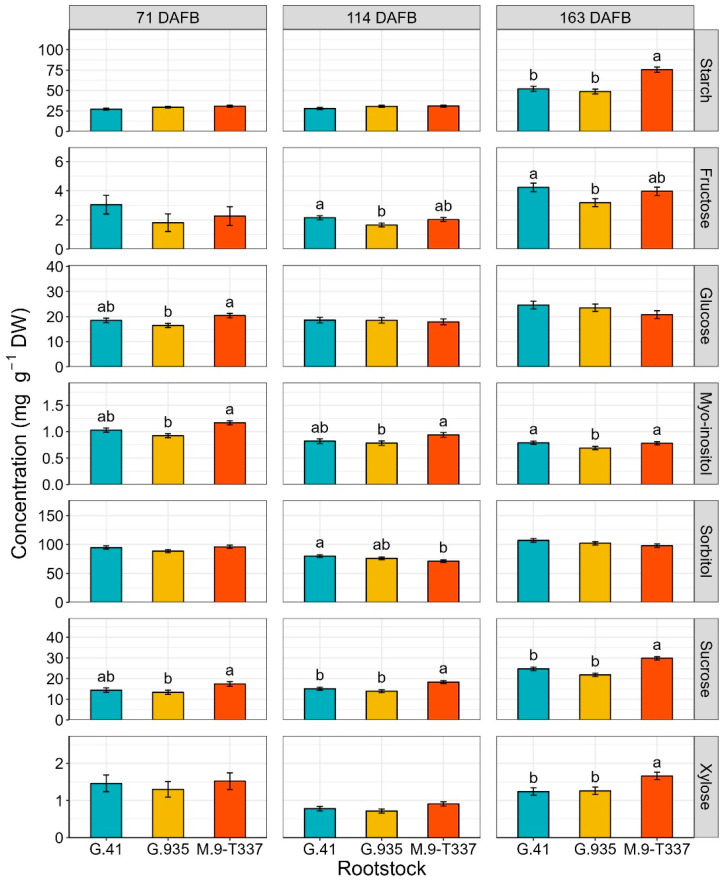
Estimated marginal means of non-structural carbohydrate concentrations (mg g^−1^ DW) (±SE, n = 12) of ‘Honeycrisp’ leaves, as affected by rootstocks ‘G.41’, ‘G.935’ and ‘M.9-T337’, under Quincy (WA) growing conditions at different time points (71, 114, 163 DAFB) throughout season 2020. From top to bottom: starch, fructose, glucose, myo-inositol, sorbitol, sucrose, and xylose. At 163 DAFB, the apples were already harvested from trees. Different letters denote significant differences (Tukey’s HSD test, *p* < 0.050) among rootstocks within each time point. Absence of letters for mean separation indicates non-significant differences.

Concerning rootstock effect on leaf starch and xylose content, a trend was observed throughout the season, with ‘G.935’ and ‘G.41’ consistently showing lower accumulation of both sugars than ‘M.9-T337’ (Figure 1). However, differences were only significant after harvest (163 DAFB).

Fructose concentration in ‘Honeycrisp’ leaves was significantly affected by rootstock at 114 DAFB (*p* = 0.032) and 163 DAFB (*p* = 0.032). ‘G.41’ was the rootstock that accumulated the most fructose on average, while ‘G.935’ accumulated the least (Figure 1). Rootstocks affected leaf glucose concentration only at 71 DAFB (*p* = 0.0123), and ‘G.935’ (16.50 mg g^−1^) had about 20% less glucose than ‘M.9-T337’ (20.40 mg g^−1^), while ‘G.41’ fell in between with 18.50 mg g^−1^ (Figure 1). Myo-inositol was affected by type of rootstock throughout the season (Figure 1). ‘G.935’ consistently recorded the lowest myo-inositol concentrations, but differences were mostly not significant compared to ‘G.41’ (Figure 1). Rootstock effect on sorbitol was significant only at 114 DAFB (*p* = 0.046), with ‘G.41’ showing higher sorbitol content than ‘M.9-T337’, but similar concentrations to ‘G.935’ (Figure 1). Rootstocks had a significant effect on leaf sucrose content throughout the season, and ‘M.9-T337’consistently exhibited the highest concentrations (Figure 1).

**Figure 2:**
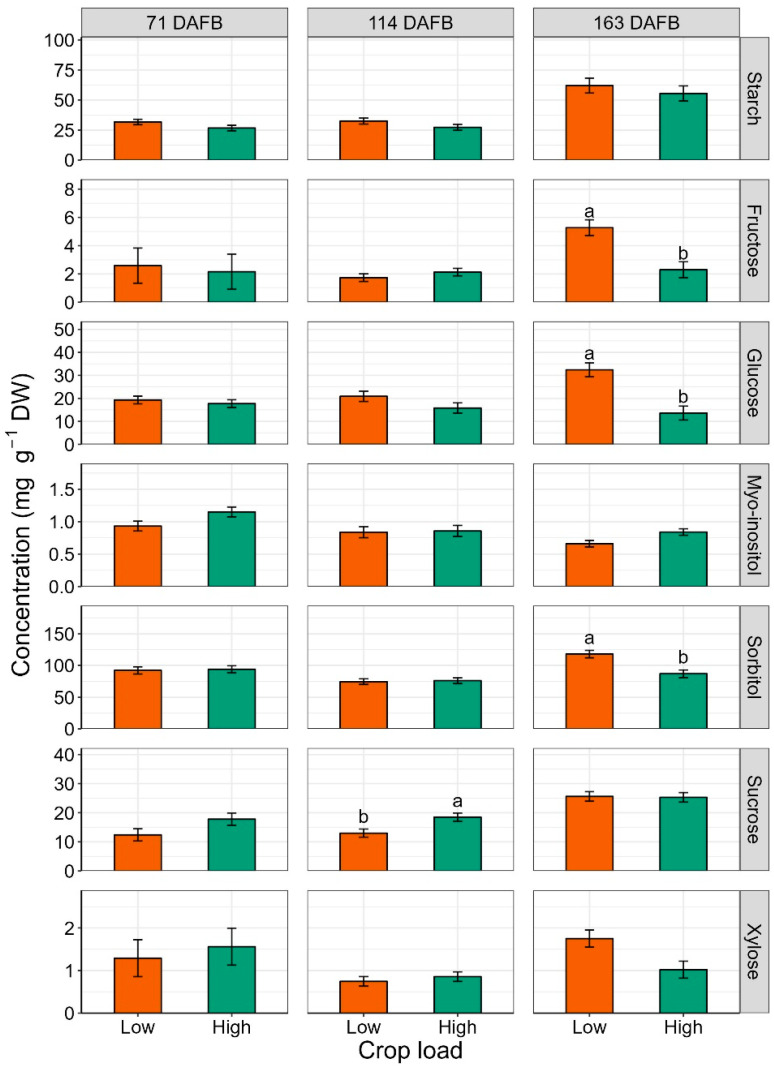
Estimated marginal means of non-structural carbohydrate concentrations (mg g^−1^ DW) (±SE, n = 18) of ‘Honeycrisp’ leaves, as affected by low and high cropping, under Quincy (WA) growing conditions at different time points (71, 114, 163 DAFB) throughout season 2020. From top to bottom: starch, fructose, glucose, myo-inositol, sorbitol, sucrose, and xylose. At 163 DAFB, the apples were already harvested from trees. Different letters denote significant differences (Tukey’s HSD test, *p* < 0.050) among rootstocks within each time point. Absence of letters for mean separation indicates non-significant differences.

Starch content was consistently higher in leaves of low-cropping trees throughout the season; however, the difference was not significant at any time point (Figure 2).

The effect of crop load on soluble carbohydrate accumulation in leaves was mainly observed after fruit removal (163 DAFB). Crop load significantly impacted fructose (*p* = 0.001), glucose (*p* = 0.003) and sorbitol (*p* = 0.019) contents (Figure 2). The highest accumulation of these sugars was found in leaves from low-cropping trees. Before harvest, crop load only had a significant impact on sucrose concentration at 114 DAFB, and the highest value (18.50 mg g^−1^) corresponded to the high crop load treatment (Figure 2).

**Figure 3:**
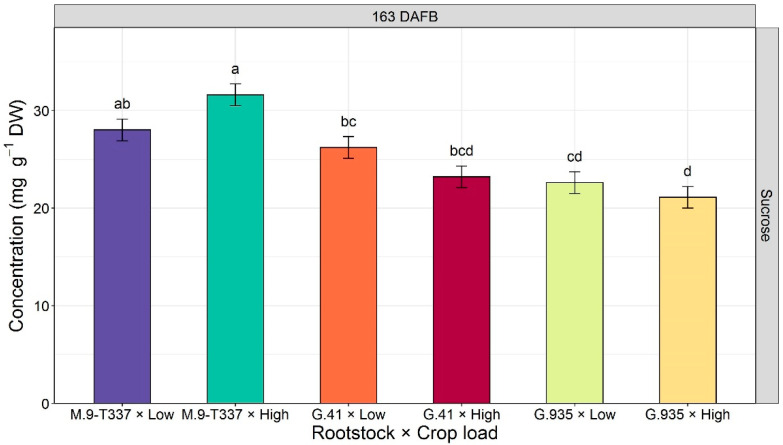
Estimated marginal means (emmeans) of rootstock and crop load interactions for leaf sucrose concentration (mg g^−1^ DW) (±SE, n = 6) of ‘Honeycrisp’ leaves at 163 DAFB (after harvest = defruited trees) under Quincy (WA) growing conditions (season 2020). Emmeans followed by different letters were significantly different at *p* < 0.050 according to Tukey’s HSD test.

The interaction between rootstock and crop load was significant only for sucrose at 163 DAFB (*p* = 0.016, Figure 3). Rootstock ‘M.9-T337’ at high crop load levels recorded the highest leaf sucrose content (31.6 mg g^−1^), while ‘G.935’ thinned to high crop load levels had the lowest (21.1 mg g^−1^, Figure 3).

### 2.3. Fruit Non-Structural Carbohydrates

**Figure 4:**
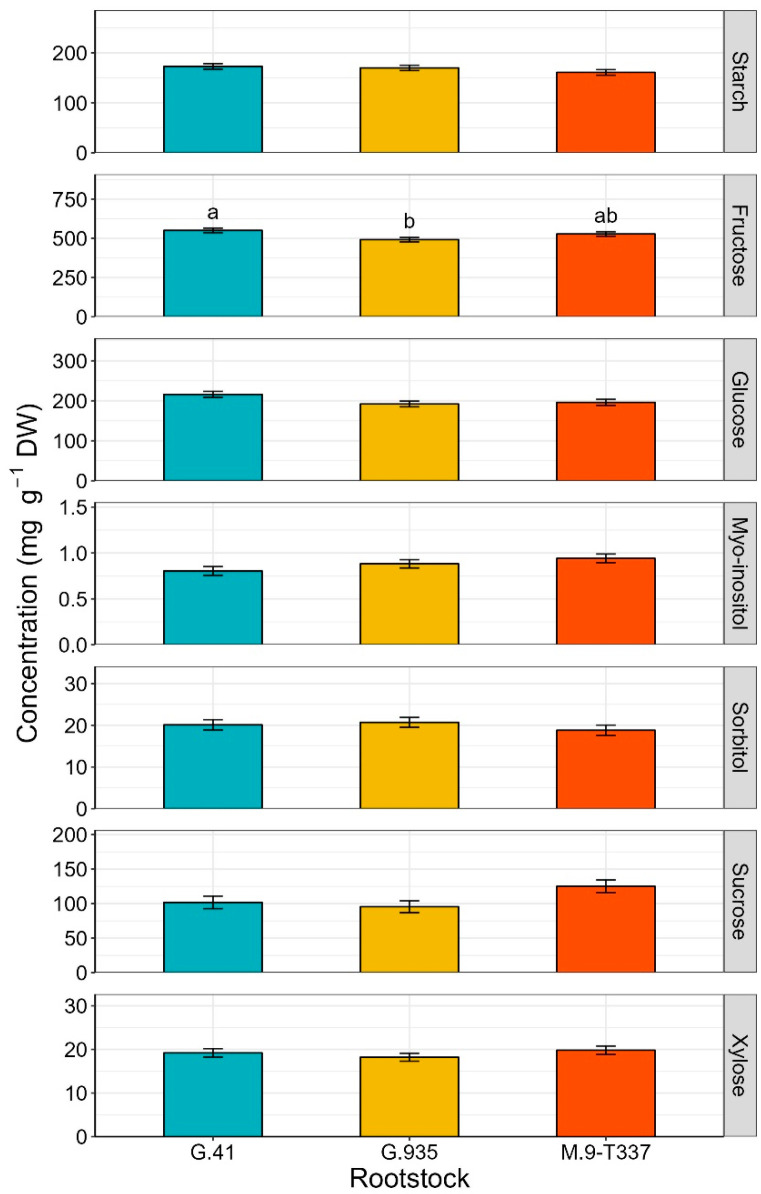
Estimated marginal mean of non-structural carbohydrate concentrations (mg g^−1^ DW) (±SE, n = 12) of ‘Honeycrisp’ fruit sampled two months postharvest, as affected by rootstocks ‘G.41’, ‘G.935’ and ‘M.9-T337’, under Quincy (WA) growing conditions. From top to bottom: starch, fructose, glucose, myo-inositol, sorbitol, sucrose, and xylose. Different letters denote significant differences (Tukey’s HSD test, *p* < 0.050). Absence of letters for mean separation indicates non-significant differences.

In apple cortex sampled two months postharvest, rootstock effect was significant on fructose concentrations only (*p* = 0.020, Figure 4). Similar to what was observed in leaves, rootstock ‘G.935’ had the lowest fructose concentration in fruit (492.0 mg g^−1^) and ‘G.41’ the highest (551.0 mg g^−1^). For all other sugars (starch, glucose, myo-inositol, sorbitol, sucrose, and xylose) rootstock effect was not significant.

**Figure 5:**
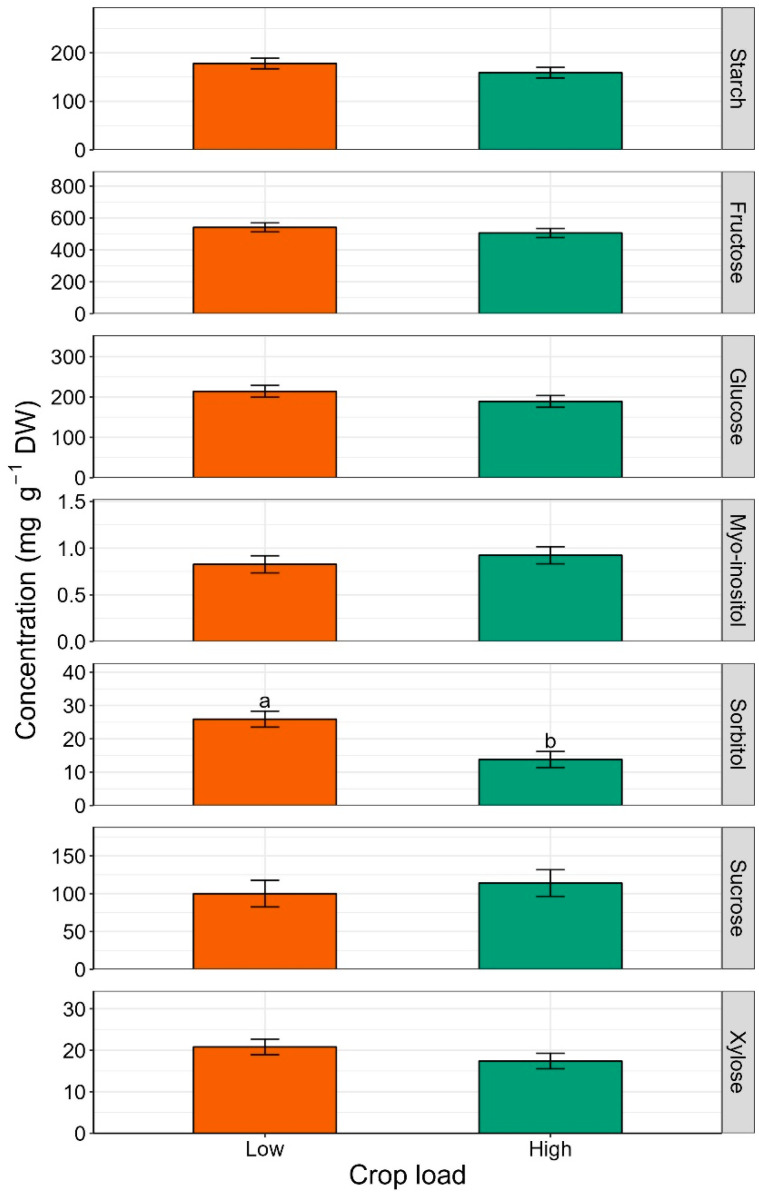
Estimated marginal means of non-structural carbohydrate concentrations (mg g^−1^ DW) (±SE, n = 18) of ‘Honeycrisp’ fruit sampled two months postharvest, as affected by low and high cropping, under Quincy (WA) growing conditions. From top to bottom: starch, fructose, glucose, myo-inositol, sorbitol, sucrose, and xylose. Different letters denote significant differences (Tukey’s HSD test, *p* < 0.050). Absence of letters for mean separation indicates non-significant differences.

Crop load significantly influenced fruit sorbitol content (*p* = 0.014, Figure 5). The low crop load treatment increased sorbitol concentration in fruit cortex by about 47%. Fruit from low-cropping trees also had a higher starch content than fruit from high-cropping trees, but the difference was not significant. Likewise, no significant differences were observed between crop load levels for fructose, glucose, myo-inositol, sucrose, and xylose contents.

### 2.4. Soluble Solids Content (SSC) and Dry Matter (DM)

**Figure 6:**
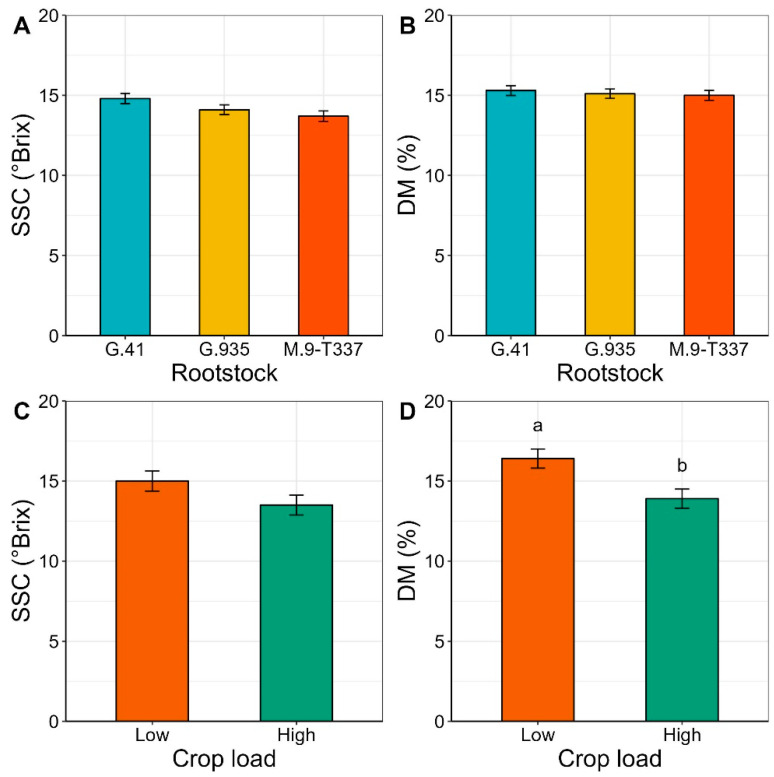
Estimated marginal means of soluble solids content (SSC, °Brix) and dry matter (DM, %) of ‘Honeycrisp’ fruit sampled two months postharvest, as affected by (**A**,**B**) rootstocks ‘G.41’, ‘G.935’ and ‘M.9-T337’ (±SE, n = 12) and (**C**,**D**) low and high crop load (±SE, n = 18), under Quincy (WA) growing conditions in season 2020. Absence of letters for mean separation indicates non-significant differences.

Rootstock effect on SSC and DM was not significant (Figure 6A,B). Crop load had a significant effect on DM, with apples from low-cropping trees showing above 16% DM on average, while apples from high-cropping trees barely reached 14% (Figure 6D).

## 3. Discussion

### 3.1. Photosynthesis and Chlorophyll Content

Positive correlations between tree size and photosynthetic performance have been reported in recent studies on apple trees [23,24], as well as on other tree fruit species (cherry [32]; pear [33,34]). In the present trial, the TCSA of ‘G.935’ was larger than that of ‘M.9-T337’, but not significantly different from ‘G.41’ (Table S1). Still, ‘Honeycrisp’ grafted on ‘G.935’ exhibited the highest Pn in all measurements taken between 98 and 129 DAFB (Table 1)**.** Previous greenhouse (non-fruiting trees) and field experiments (fruiting trees) grouped ‘G.935’ together with vigorous rootstocks that enhanced scions’ (‘Fuji’ and ‘Honeycrisp’) photosynthesis and transpiration [25,35].

Although here, based on TCSA measurements, ‘G.935’ was not significantly more vigorous than ‘G.41’, it could be that the graft union between ‘G.935’ and ‘Honeycrisp’ did not exhibit altered xylem anatomy [36] (typical of graft unions between scions and dwarfing rootstocks), which has been associated with poor hydraulic conductivity and subsequently reduced photosynthetic capacity [25,37].

Interestingly, after fruit harvest (162 DAFB), ‘G.935’ showed the lowest E and gs values in the present experimental conditions, suggesting that the presence of fruit notably enhanced the photosynthetic potential of this rootstock. Ci is an indicator of the CO_2_ available for Pn [38], and in general the two parameters are positively correlated (Pn increases with increasing Ci) [39,40,41]. However, in this experiment ‘G.935’ displayed the highest Pn and lowest Ci, while ‘G.41’ and ‘M.9-T337’ had lower Pn values compared to ‘G.935’ but similar or higher Ci and gs values (Table 1). These observations might support a non-stomatal limitation of Pn, i.e., the lower Pn of ‘G.41’ and ‘M.9-T337’ might not be induced by stomatal closure and Ci limitation.

Concerning crop load effect on fluorescence, Φ_PSII_ values were significantly different between crop loads at 115 and 129 DAFB, and high crop load recorded the highest means (Table 1), thus pointing to a higher use efficiency of photochemical transports for photosynthesis, mitochondrial respiration, photorespiration, and alternative electron pathways [42]. As expected, ETR, a parameter directly proportional to Φ_PSII_ and representing the flux of electrons exiting PSII, mirrored the pattern of Φ_PSII_, and the higher values found for high-cropping trees are indicative of a higher leaf photosynthetic potential [23]. Although in apple, increasing crop load levels are commonly associated with increased photosynthetic efficiency—up to reaching a plateau [30,43]—evidence supporting the opposite trend is also found in the literature [31]. The gas exchange and fluorescence results indicate that low crop load leads to a decline of gas exchanges in ‘Honeycrisp’ apple trees, which probably caused a feedback inhibition for sink limitation [44]. On ‘Gala’, grafted on ‘M.26’ rootstock, the feedback inhibition of photosynthesis resulted in a reduced activity of RuBisCO on low crop load samples [21]. Fluorescence parameter efCO_2_ was generally higher in leaves of ‘Honeycrisp’ grafted on ‘G.935’, suggesting that this rootstock enhanced photosynthetic performance. This was consolidated by the fact that ‘G.935’ leaves often had the lowest electron use efficiency of non-net carboxylative processes (efNC), thus pointing to reduced activity of these processes. In ‘G.41’ and ‘M.9-T337’, net photosynthesis was not limited by the electron flux (ETR), as well as the reduction in Pn, which did not feedback limit the electron transport chain. In severe stress conditions, like strong water stress and/or high temperature, the absorbed energy is not used for carbon fixation (one of the electron user processes), but is diverted to non-photochemical processes (i.e., the xanthophyll cycle), thereby reducing the electron transport rate exiting from PSII. In the present study, the electron transport activity was not affected, and the energy not used for carbon fixation was instead funneled to alternative electron transport like photorespiration, water–water cycle, and cyclic transport around PSI. This behavior was also observed in peach, pear, grapevine, and apple trees subjected to moderate stress [45,46]. Overall, the information gathered from the gas exchange and chlorophyll fluorescence analyses suggested that ‘Honeycrisp’ grafted on ‘G.935’ had the highest photosynthetic performance. Tworkoski and Fazio (2011) [25] found that ‘Fuji’ grafted on ‘G.935’ had higher photosynthesis and transpiration rates compared to more dwarfing rootstocks, and similar observations were reported by Lordan et al. (2017) [35] on ‘Honeycrisp’.

Regarding the effect of rootstock on leaf chlorophyll content, previous studies conducted in cherry and apple [28,32] reported higher chlorophyll concentrations in leaves of dwarfing rootstocks. However, in the present study, no pattern was observed for SPAD values between rootstocks after 65 DAFB (Table 1). In the case of crop load, contrasting results can be found in the literature about the effect of crop load on leaf chlorophyll content. While Wünsche et al. (2005) [29] reported that chlorophyll concentration increased with increasing crop load levels, Ding et al. (2017) [31] observed the opposite trend in their experiment, with low-cropping inducing the highest SPAD values. Different apple cultivars, as well as different rootstocks (‘Braeburn’/‘M.26’ and ‘Red Fuji’/‘M.26’/*Malus hupehensis* Rehd.), were used in the two studies, which could potentially account for the differences in chlorophyll content, as well as for the abovementioned different photosynthetic performance. In the present trial, leaves from high-cropping trees in general had higher SPAD values than leaves from low-cropping trees, but the differences were not significant (Table 1).

### 3.2. Leaf Non-Structural Carbohydrates

Analysis of non-structural carbohydrates revealed that the best discrimination between treatments for sugar accumulation in leaves was after harvest (163 DAFB, Figure 1 and Figure 2). In a previous rootstock trial, Brown et al. (1985) [47] noticed the same result regarding carbohydrate contents of above- and below-ground parts of ‘Redchief’ and ‘Northern Spy’ apple trees on rootstocks ‘M.9’ and ‘MM.111’. This could be related to carbohydrate concentrations fluctuating during the season due to metabolic activities. Toward the end of the season, and especially after fruit removal (i.e., after 162 DAFB), the metabolic slowdown may stabilize sugar content in tissues, enabling the detection of differences in carbohydrate accumulation. At the end of the season, starch concentration was the highest in leaves of ‘Honeycrisp’ grafted on ‘M.9-T337’ (Figure 1), the most dwarfing rootstock in the trial. Accumulation of starch in both rootstock and scion tissues has been documented when dwarfing or semi-dwarfing rootstocks were used [23,27]. Previous studies reported a decrease in soluble sugars, including glucose, fructose, sorbitol, and myo-inositol, concomitant with starch accumulation in rootstocks with high dwarfing potential [23,27]. Nevertheless, Samuolienė et al. (2016) [28] found that ‘P.22’, a super-dwarfing rootstock, induced accumulation of glucose, fructose, and sorbitol in apple cultivar ‘Ligol’ leaves. In the present experiment, it was not possible to make a univocal conclusion about the rootstock effect on leaf-soluble sugars of ‘Honeycrisp’ scions because of the observed variability. The impact of crop load treatments on leaf carbohydrates was observed almost exclusively at the end of the season (Figure 2). In a study on peach cv. ‘Yanfengyihao’, the fruit removal resulted in an accumulation of starch and sorbitol in leaves, confirming our results for sorbitol at 163 DAFB [44]. Low crop load clearly induced a general increase in soluble carbohydrates, specifically fructose, glucose, and sorbitol, in accordance with what was observed by Wünsche et al. (2005) [29] on ‘Braeburn’/’M.26’. Contrary to that study and observations on peach and pear [44,48], here leaves from low-cropping trees did not undergo a significant increase in starch content. This is remarkable since low cropping in ‘Honeycrisp’ has often been associated with starch granules buildup in leaves and leaf yellowing, a physiological disorder named zonal leaf chlorosis [49,50]. Leaves affected by this disorder also showed decreased CO_2_ assimilation compared to healthy ones [51]. A linear relationship between leaf starch content and photosynthetic decline has been reported, supporting the theory that end-product accumulation triggers mechanisms (RuBisCO decreased activity, PSII damage, stomatal closure, leaf chlorophyll degradation) that lead to carbon assimilation inhibition [52,53,54]. Here, neither starch overaccumulation nor decreased chlorophyll content were observed in leaves of low-cropping trees, suggesting that the lower photosynthetic efficiency could have been induced just by the accumulation of soluble sugars. Araya et al. (2006) [55] reported in their study on *Phaseolus vulgaris* L. that the photosynthetic genes could be inhibited by soluble sugars [56], in particular glucose level, potentially reducing photosynthesis (at saturating CO_2_ concentration, A_max_) in source leaves. This could be a possible explanation for the higher postharvest (163 DAFB) glucose concentration in ‘Honeycrisp’ leaves and the almost zero Pn at 162 DAFB (Figure 2 and Table 1). Wünsche et al. (2005) [29] postulated that at mid-season, after the termination of shoot growth in low sink-trees, the photoassimilates might be utilized for vegetative growth (i.e., trunk growth) and redirected to other sinks that were too minor to retain the high Pn capacity. After harvest, (former) fruiting trees recorded lower photosynthesis than at mid-season, but still relatively high, probably to satisfy the needs of other sinks to prepare for entering dormancy [19]. The presence of a trend in chlorophyll and starch content but a lack of significance could mean that more replications are required for thorough discrimination of crop load effects.

### 3.3. Fruit Non-Structural Carbohydrates, Soluble Solids Content (SSC), and Dry Matter (DM)

The effect of both rootstock and crop load became less evident when considering fruit carbohydrates, as significant differences were observed only for fructose and sorbitol. Consistent with what was reported for leaves at the end of the season, fruit from ‘G.935’ had the lowest fructose concentration and fruit from ‘G.41’ the highest (Figure 4). High fructose concentrations could be explained by the higher accumulation capacity of this sugar in vacuoles or by higher conversion rates of sorbitol into fructose. Instead, lower fructose content, as observed in ‘G.935’, tissues may indicate faster consumption or conversion of fructose into other sugars to sustain metabolic functions in our experimental conditions. The highest sorbitol values recorded for fruit from low-cropping trees reflected the high content recorded for leaves, potentially suggesting that reduced competition among fruits on low-cropping trees leads to an increased fruit sink strength for sorbitol.

Regarding fruit quality parameters, the increase in DM observed in ‘Honeycrisp’ fruit from the low crop load treatment is consistent with previous reports on ‘Honeycrisp’ [57], ‘Braeburn’ [29], and ‘WA 38’ [58]. Similar to the higher content of sorbitol in apples from low-cropping trees, higher DM accumulation could be explained by the increased sink strength of fruit in a situation of reduced competition for assimilates [59,60]. In the present experiment, neither rootstock nor crop load treatments exerted a significant effect on fruit SSC. Notably, Serra et al. (2016) [57] observed an increase in SSC of ‘Honeycrisp’ apples from trees thinned to 4.7 fruit cm^−2^ trunk cross-sectional area (TCSA) compared to trees thinned to 7.5 fruit cm^−2^ TCSA. Instead, at similar crop load levels (4.1 and 7.8 fruit cm^−2^ TCSA), no significant differences in SSC were found for fruit of apple cultivar ‘WA 38’ [58]. In that trial [58], higher SSC in ‘WA 38’ apples thinned to 2.1 fruit cm^−2^ was found when compared to SSC in 4.1, 6.0 and 7.1 fruit cm^−2^ treatments. Moreover, ‘WA 38’ dry matter (%) significantly declined in 6.0 and 7.1 fruit cm^−2^ in comparison to 2.1 and 4.1 fruit cm^−2^. Lastly, significant differences in SSC were observed in ‘Royal Gala’ trees between all crop load treatments (3, 4, and 5 fruit cm^−2^ limb cross-sectional area, LCSA), with the highest crop load presenting an average value of 1.1 °Brix lower than the lowest crop load [61]. These results suggest that the effect of crop load on fruit SSC could also depend on other factors, such as apple cultivar, growing conditions, training system, timing of thinning, and additional agronomic practices [62,63].

## 4. Materials and Methods

### 4.1. Experimental Site and Tree Selection

The experiment was conducted in 2020 in a ‘Cameron Select^®^ Honeycrisp’ (HC) commercial orchard located in a semiarid environment (Quincy, WA, USA) with silt loam soil. Trees were planted in 2013 (North–South oriented rows) with a spacing of 0.6 m × 3.7 m (4504 trees ha^−1^) and were trained on a 6-wire V-trellis. Starting from 2018, the orchard management was carried out according to the USDA organic regulations. Irrigation was administered through a drip system in the rows and micro-sprinklers between rows.

**Figure 7:**
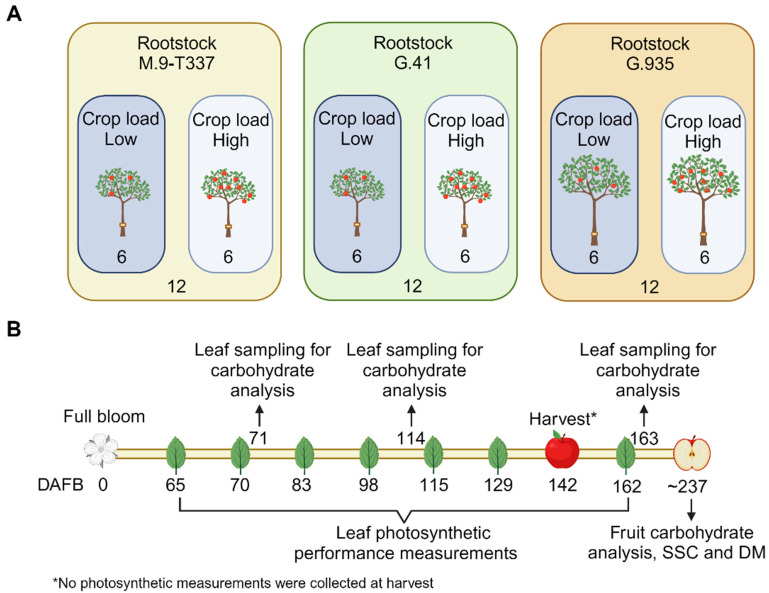
Schematic diagram depicting (**A**) the experimental design with three rootstocks and two crop load levels for ‘Honeycrisp’ scion and (**B**) the timeline of the experiment in 2020. The numbers 6 and 12 in panel A represent the number of trees per rootstock thinned to low and high crop load, and the total number of ‘Honeycrisp’ trees per rootstock included in the experiment. Abbreviations: DAFB = days after full bloom; SSC = soluble solids content; DM = dry matter.

‘Cameron Select^®^ Honeycrisp’ trees grafted onto ‘M.9-T337’, ‘G.41’, and ‘G.935’ rootstocks were utilized for this experiment (Figure 7A). Experimental trees were selected in Spring of 2020 by measuring trunk diameters and counting flower clusters. Trunk diameters were measured at 10 cm above the graft union using a digital caliper and were used to calculate trunk-cross sectional area (TCSA). After counting flower clusters per tree, 36 trees in total were selected (12 trees per rootstock).

Six weeks after full bloom (21 April 2020), 18 trees (6 per rootstock) were hand-thinned to a low crop load range, averaging 3.3 fruit cm^−2^ TCSA, and the other 18 trees (6 per rootstock) to a high crop load range (Figure 7A), with an average of 9.4 fruit cm^−2^ TCSA (Table S1) (3 rootstocks × 2 crop load levels × 6 reps = 36 trees in total).

### 4.2. Photosynthesis and Chlorophyll Content

Leaf gas exchange, chlorophyll fluorescence, and chlorophyll content were assessed on sunny days, 65, 70, 83, 98, 115, 129, and 162 days after full bloom (DAFB) (Figure 7B). Environmental conditions (wind speed, solar radiation, air temperature, and relative air humidity, Table S3) were recorded every 5 min with an ATMOS 14 weather station located within the experimental rows and equipped with an EM50 datalogger (Meter Group, Pullman, WA, USA).

Gas exchange measurements were taken from about 10:30 a.m. to 12:30 p.m. on 2 mature, healthy, and sun-exposed leaves per tree, and from 3 trees per rootstock-crop load combination located on the East side of the rows. An infrared gas analyzer (LI-6400XT, LI-COR, Lincoln, NE, USA) equipped with a 2 cm^2^ leaf chamber with a LED light source was used to measure carbon assimilation (Pn, μmol m^−2^ s^−1^), stomatal conductance (gs, mol m^−2^ s^−1^), transpiration rate (E, mmol m^−2^ s^−1^), and intercellular CO_2_ concentration (Ci, μmol mol^−1^ air). Leaf chamber temperature (°C) and photosynthetic photon flux density (PPFD, μmol m^−2^ s^−1^) were set equal to environmental conditions measured using the external quantum sensor mounted on the LI-6400XT head at each time point and maintained stable during measurements (Table S3). Reference CO_2_ concentration, flow rate, and leaf fan speed were set at 400 μmol mol^−1^, 400 μmol s^−1^, and fast for all time points, respectively.

Chlorophyll fluorescence parameters (*F*_0_’, *F_m_*’ and *F_s_*) on light-adapted leaves were recorded simultaneously to gas exchange using the integrated leaf chamber fluorometer. The following variables were derived from the fluorescence parameters measured [42,64,65,66]:

maximum efficiency of PSII in the light *F_v_’*/*F_m_’* = (*F_m_*’ − *F_0_*)/*F_m_*’ (1)

effective quantum yield of PSII of a light adapted leaf Φ_PSII_ = (*F_m_*’ − *F_s_*)/*F_m_*’ (2)

electron transport rate ETR = Φ_PSII_ × PPFD × 0.5 × 0.87 (3)

where 0.5 accounts for the fact that two photons move one electron and 0.87 is the average leaf absorbance.

net photosynthesis expressed as electron transport rate J_CO_2__ = Pn × 4 (4)

where 4 represents the number of electrons used to fix 1 molecule of CO_2._

residual absorbed energy that is used for non-net carboxylative processes (e.g., photorespiration, dark respiration, and electron transports) J_NC_ = ETR − J_CO_2__ (5)

electron use efficiency of photosynthesis efCO_2_ = J_CO_2__/ETR (6)

electron use efficiency of non-carboxylative processes efNC = J_NC_/ETR (7)

Leaf chlorophyll content was assessed using a chlorophyll meter (SPAD502Plus, Konica Minolta, Tokyo, Japan) on the same trees selected for photosynthesis measurements. SPAD measurements were taken on 2 mature, healthy leaves per tree.

### 4.3. Non-Structural Leaf Carbohydrate Determination

Leaves for non-structural carbohydrate analysis were sampled from the orchard in the morning at 71, 114, and 163 DAFB (Figure 7B). The postharvest leaf sampling [21,47,48] was planned to assess rootstock and crop load effects on photosynthesis and carbohydrate accumulation after fruit (sink) removal. Eight mature, healthy leaves were sampled from each experimental tree placed in a cooler with ice packs as they were collected and transported to the laboratory. When in the laboratory, leaves were immediately washed with deionized water, blotted, and frozen with liquid nitrogen. Samples were then stored at –80 °C until freeze-drying in a lyophilizer (FreeZone 12 plus, Labconco, Kansas City, MO, USA). Dried samples were ground with an analytical mill (IKA A 11 Basic, IKA Works Inc., Wilmington, NC, USA) and stored at room temperature (RT) until further analysis. Powdered samples were accurately weighed (~100 mg), and starch content was analyzed using a total starch assay kit (Total Starch HK Assay Kit, Megazyme, Bray, Ireland) and following the manufacturer’s procedure. The sample solutions used for the spectrophotometric assays, conducted on an Agilent Cary 60 UV-Vis (Agilent, Santa Clara, CA, USA), were concentrated 0.1 times. An aliquot of each ground sample was used for soluble carbohydrates (fructose, glucose, myo-inositol, sorbitol, sucrose, and xylose) analysis. Extraction was carried out following the method by Lee et al. (2008) [67], with a few modifications. Powdered apple leaf tissue was weighed, resuspended in 1000 μL of extraction solvent (methanol:2-propanol:water, 5:2:2 *v*/*v*/*v*), shaken at RT for 10 min (Vortex-Genie-2T, Scientific Industries, Bohemia, NY, USA), and sonicated at RT for 10 min (Branson 5510 sonication bath, Branson Ultrasonics Corp, Brookfield, CT, USA). The extracts were centrifuged at 21,000× *g* for 15 min at RT. Ten μL of the supernatants were diluted to 500 μL with the same extraction solvent, and 0.5 μg of salicylic acid-d6 internal standard (C/D/N Isotopes, Pointe-Claire, QC, Canada) was added to the extract before vacuum-drying (Eppendorf Vacufuge Plus Concentrator, Eppendorf, Hamburg, Germany) of 50 μL aliquots. External calibration curves were obtained with diluted authentic standards. Seven calibration points were used, ranging over 0.8–48.0 μg mL^−1^ (fructose), 0.8–24.0 μg mL^−1^ (glucose and sucrose), 0.04–1.20 μg mL^−1^ (myo-inositol), 2–24 μg mL^−1^ (sorbitol) and 0.01–24.00 μg mL^−1^ (xylose). The dry residues were suspended in 5 μL *O*-methoxylamine hydrochloride (30 mg mL^−1^ in pyridine; Sigma, St. Louis, MO, USA) and incubated for 90 min at 30 °C and 1000 rpm (Thermomixer R, Eppendorf). Subsequently, derivatization was performed with 45 μL of N-methyl-N-(trimethylsilyl) trifluoroacetamide (MSTFA) with 1% trimethylchlorsilane (TMCS) (Thermo Fisher Scientific, Waltham, MA, USA) for 30 min at 37 °C and 1000 rpm. Gas chromatography–mass spectrometry analysis was carried out following the protocol reported in Attaran et al. (2020) [68]. Peak alignment and spectrum comparisons were carried out with the Statistical Compare feature of the ChromaTOF^®^ software v.4.50.8.0 (LECO, St. Joseph, MI, USA). Concentrations of target metabolites in dry weight were calculated based on external calibration curves and the accurate weight of tissue used for extraction.

### 4.4. Yield and Fruit Sorting

All fruits of all experimental trees were harvested at commercial maturity (142 DAFB, starch index ranging from 4 to 6 in a 6-point scale). The number of apples harvested from each tree and the yield (kg tree^−1^) were recorded. Based on this data, crop load at harvest (no. fruit cm^2^ TCSA) and average fruit weight (g) were determined. Averages of tree performance parameters for each rootstock, crop load level, and rootstock-crop load combinations are reported in Table S2. Soon after harvest, all harvested apples were stored in a cold room at ~1 °C and regular atmosphere. Two months later, all fruits were sized with a customized sizer [58]. Apples in the 70–85 mm diameter range were assessed for I_AD_, a non-destructive ripening index [69]. For each fruit, two DA-meter (Turoni s.r.l., Forlì, Italy) readings were taken along the equatorial line, on the sun and shade cheeks, and averaged.

### 4.5. Non-Structural Apple Fruit Carbohydrate, Soluble Solids Content (SSC) and Dry Matter (DM) Determination

For carbohydrate analysis, two months after harvest, eight apples per experimental tree (3 rootstocks × 2 crop load levels × 6 reps × 8 apples = 288 apple fruits in total) were selected in the 70–85 mm diameter range and with I_AD_ values between mean ± standard deviation. Apples were left at room temperature for 24 h before processing to re-equilibrate. An equatorial slice of about 1 cm thick was sampled from each fruit, peel, and seed cavities were removed, and the slice was cut into small dice. Apples from the same tree were pooled together representing one rep (6 reps per each rootstock-crop load treatment), then frozen with liquid nitrogen and stored at –80 °C prior to freeze-drying. Further sample processing for starch assay and soluble carbohydrate analysis were conducted following the same protocol described for leaves (Section 4.4), with the exception that the final solutions used for the spectrophotometer assay were not concentrated.

While preparing the samples for carbohydrate analysis, two slices adjacent to the equatorial line were cut from each apple and used for SSC and DM assessment. SSC and DM were measured following Anthony et al. (2019) [58], and expressed as °Brix and percentage, respectively.

### 4.6. Statistical Analyses

Data were analyzed using R version 4.0.2 (R-Core-Team, 2020). Names of R packages and functions are reported as ‘package name:function name’ within round brackets. Photosynthesis variables, SPAD measurements, and leaf carbohydrate concentrations were analyzed separately for each measurement/sampling day. Linear mixed models (nlme:lme [70]) were built to assess rootstock and crop load effects on photosynthesis variables, DM and SSC. Rootstock and crop load treatment (low/high) were included in the models as factors, the actual crop load recorded at harvest was treated as a covariate, and the replicate (tree) as a random effect. A linear model was fit for carbohydrate concentration analysis, including rootstock and crop load range as factors and actual crop load as a covariate. Analysis of covariance (ANCOVA) with type III sums of squares was conducted (car:Anova [71]), and differences were considered significant at *p* < 0.050. Means that were significantly different were separated using Tukey’s HSD test (emmeans:emmeans [72]). All means and standard errors reported are estimated marginal means and estimated marginal standard errors.

## 5. Conclusions

Low crop load reduced the photosynthetic performance of ‘Honeycrisp’ trees, possibly due to the increase in leaf fructose, glucose, and sorbitol concentrations. In the present experimental conditions, ‘Honeycrisp’ grafted on ‘G.935’ exhibited higher carbon assimilation capacity and reduced activity of non-carboxylative processes, confirming previous reports for this rootstock. Further investigation into the anatomy of the graft union and hydraulic potential measurements could clarify the mechanism/s behind the performance of ‘G.935’. Crop load and rootstock treatments did not significantly affect the carbohydrate composition of the fruit, except for fructose and sorbitol, suggesting that the pathways underlying the distribution and concentration of these sugars could be sensitive to changes in rootstock and crop load treatments. In agreement with previous studies, here, a higher DM accumulation was found in fruit from low-cropping ‘Honeycrisp’ trees, thus remarking the key role of crop load in the allocation of photoassimilates.

## Figures and Tables

**Table 1:** Estimated marginal means (emmeans) of leaf gas exchange variables, chlorophyll fluorescence variables and SPAD measurement for ‘Honeycrisp’ grafted onto rootstocks ‘G.41’, ‘G.935’, ‘M.9-T337’ (n = 6), thinned to low and high crop load levels (n = 9), and interactions between rootstock and crop load treatments (n = 3), under Quincy (WA) growing conditions at different time points throughout season 2020. Emmeans followed by different letters were significantly different at *p* < 0.050 according to Tukey’s HSD test. Absence of letters for mean separation indicates non-significant differences.

DAFB	Experimental Factor	Gas Exchange Variable ^1^				Fluorescence Variable ^2^							SPAD ^3^
		Pn	Ci	E	g_s_	J_CO_2__	efCO_2_	*Fv’/Fm’*	Φ_PSII_	ETR	J_NC_	efNC	
65	Rootstock (R)												
	G.41	5.6	266	2.42	0.084	22.2	0.205	0.406	0.122	110.0	87.4	0.795	46.1 b
	G.935	7.2	255	3.02	0.093	28.9	0.238	0.381	0.134	119.0	90.4	0.762	49.5 a
	M.9-T337	7.5	265	2.99	0.109	30.1	0.252	0.398	0.130	117.0	86.4	0.748	44.2 b
	*p* < 0.050	0.715	0.701	0.784	0.792	0.716	0.827	0.854	0.777	0.814	0.861	0.827	<0.001
	Crop load (C)												
	Low	4.5	253	1.81	0.054	18.0	0.219	0.359	0.024	77.9	56.2 a	0.781	44.7
	High	9.1	271	3.80	0.136	36.2	0.245	0.431	0.172	152.4	119.8 b	0.755	48.6
	*p* < 0.050	0.499	0.100	0.430	0.391	0.499	0.894	0.559	0.060	0.069	0.001	0.894	0.111
	R × C												
	*p* < 0.050	0.282	0.055	0.763	0.713	0.282	0.158	0.063	0.406	0.411	0.296	0.158	0.161
70	Rootstock (R)												
	G.41	4.4	292 a	1.63	0.060	17.6	0.141 b	0.340	0.109	99.8	82.2	0.859 a	48.0
	G.935	10.3	181 b	2.42	0.090	41.2	0.340 a	0.379	0.130	119.5	78.2	0.660 b	45.0
	M.9-T337	7.3	274 a	2.65	0.099	29.0	0.228 ab	0.439	0.120	111.3	82.3	0.772 ab	47.0
	*p* < 0.050	0.160	<0.001	0.532	0.572	0.160	0.012	0.403	0.628	0.607	0.903	0.012	0.124
	Crop load (C)												
	Low	5.6	222	1.09	0.039	22.3	0.232	0.278	0.087	80.5	58.2	0.768	47.2
	High	9.1	276	3.37	0.127	36.3	0.241	0.494	0.152	139.8	103.6	0.759	46.2
	*p* < 0.050	0.669	0.442	0.350	0.373	0.669	0.959	0.244	0.254	0.255	0.093	0.959	0.805
	R × C												
	*p* < 0.050	0.424	0.133	0.565	0.596	0.425	0.197	0.918	0.520	0.460	0.585	0.197	0.527
83	Rootstock (R)												
	G.41	14.3	238 b	3.82	0.172	57.4	0.399 a	0.444	0.177	143.0	85.3 b	0.601 b	48.6
	G.935	13.7	258 a	4.14	0.191	54.9	0.341 ab	0.466	0.198	158.0	103.5 ab	0.659 ab	46.3
	M.9-T337	12.0	265 a	3.82	0.179	48.1	0.295 b	0.489	0.201	162.0	113.5 a	0.705 a	48.0
	*p* < 0.050	0.510	<0.001	0.876	0.882	0.510	0.001	0.482	0.315	0.366	0.002	0.001	0.170
	Crop load (C)												
	Low	9.0	225 b	1.85 b	0.087	36.1	0.295	0.403	0.156	125.0	88.6	0.705	47.9
	High	17.7	282 a	6.00 a	0.275	70.8	0.395	0.530	0.228	184.0	113	0.605	47.4
	*p* < 0.05	0.092	0.002	0.032	0.055	0.092	0.139	0.174	0.109	0.108	0.238	0.139	0.904
	R × C												
	*p* < 0.050	0.541	0.894	0.830	0.889	0.541	0.377	0.551	0.712	0.744	0.940	0.377	0.106
98	Rootstock (R)												
	G.41	2.3 b	308	1.92	0.054	9.3 b	0.084 b	0.302	0.149	107.0	98.0	0.917 a	48.3
	G.935	6.7 a	223	2.53	0.070	26.6 a	0.205 a	0.348	0.179	128.0	101.5	0.795 b	48.9
	M.9-T337	3.2 ab	292	1.77	0.051	12.9 ab	0.123 ab	0.313	0.144	103.0	90.4	0.877 ab	48.0
	*p* < 0.050	0.005	0.506	0.494	0.596	0.005	0.004	0.500	0.345	0.365	0.788	0.004	0.849
	Crop load (C)												
	Low	0.3 b	280	0.51	0.011	1.3 b	0.061	0.259	0.108	80.7	79.8	0.939	48.2
	High	7.8 a	268	3.64	0.106	31.2 a	0.213	0.383	0.206	145.0	113.5	0.787	48.6
	*p* < 0.050	0.041	0.951	0.075	0.064	0.041	0.107	0.251	0.145	0.189	0.416	0.107	0.922
	R × C												
	*p* < 0.050	0.788	0.946	0.701	0.708	0.788	0.287	0.500	0.575	0.641	0.475	0.287	0.215
115	Rootstock (R)												
	G.41	10.2 b	280 ab	2.50	0.160	40.7 b	0.284 b	0.529	0.269	144.0	103.3	0.716 a	47.4
	G.935	12.8 a	260 b	2.85	0.175	51.4 a	0.370 a	0.581	0.266	143.0	91.4	0.630 b	48.2
	M.9-T337	9.7 b	293 a	2.53	0.179	38.8 b	0.293 b	0.562	0.255	137.0	98.5	0.707 a	47.1
	*p* < 0.050	< 0.001	< 0.001	0.489	0.714	< 0.001	0.002	0.160	0.735	0.794	0.357	0.002	0.805
	Crop load (C)												
	Low	6.7 b	261	1.19 b	0.080 b	26.8 b	0.283	0.519	0.205 b	111.0 b	84.0	0.717	46.8
	High	15.1 a	294	4.06 a	0.263 a	60.5 a	0.348	0.595	0.322 a	172.0 a	111.0	0.652	48.4
	*p* < 0.050	< 0.001	0.102	0.001	0.004	< 0.001	0.341	0.295	0.016	0.019	0.216	0.341	0.698
	R × C												
	*p* < 0.050	0.003	0.469	0.036	0.073	0.002	0.003	0.092	0.375	0.418	0.077	0.003	0.324
129	Rootstock (R)												
	G.41	7.8 b	303 a	2.76	0.154	31.2 b	0.268 ab	0.500	0.151	118.0	86.7	0.732 ab	47.3
	G.935	12.3 a	256 b	3.08	0.171	49.3 a	0.344 a	0.498	0.183	141.0	91.8	0.656 b	47.2
	M.9-T337	8.3 b	293 a	2.48	0.159	33.4 b	0.265 b	0.573	0.166	129.0	95.1	0.735 a	49.0
	*p* < 0.050	< 0.001	< 0.001	0.412	0.840	< 0.001	0.019	0.058	0.062	0.082	0.604	0.022	0.429
	Crop load (C)												
	Low	4.5 b	243 b	0.28 b	0.010 b	18.2 b	0.256	0.459	0.114 b	89.1 b	70.9	0.744	47.0
	High	14.5 a	325 a	5.27 a	0.312 a	57.5 a	0.329	0.588	0.219 a	169.3 a	111.6	0.671	48.7
	*p* < 0.050	< 0.001	0.002	< 0.001	< 0.001	< 0.001	0.330	0.154	0.003	0.004	0.059	0.385	0.658
	R × C												
	*p* < 0.050	0.818	0.142	0.291	0.351	0.818	0.839	0.583	0.957	0.943	0.986	0.805	0.767
162 (postharvest)	Rootstock (R)												
	G.41	5.7	292 ab	1.46 ab	0.098 ab	22.8	0.239	0.595	0.145	88.8	66.0	0.761	49.4
	G.935	4.5	241 b	0.99 b	0.056 b	18.0	0.222	0.580	0.142	86.9	68.9	0.778	48.5
	M.9-T337	3.3	337 a	1.88 a	0.121 a	13.2	0.135	0.537	0.161	98.0	84.8	0.865	49.4
	*p* < 0.050	0.427	0.012	0.015	0.013	0.428	0.254	0.275	0.697	0.719	0.256	0.254	0.683
	Crop load (C)												
	Low	0.0 b	313	0.68	0.048	0.0 b	0.000 b	0.493	0.159	84.5	98.2	1.064 a	46.8
	High	9.1 a	267	2.21	0.136	36.2 a	0.461 a	0.649	0.140	97.9	48.3	0.539 b	51.4
	*p* < 0.050	0.036	0.579	0.053	0.126	0.036	0.002	0.099	0.745	0.716	0.105	0.002	0.122
	R × C												
	*p* < 0.050	0.600	0.446	0.558	0.624	0.600	0.478	0.088	0.969	0.936	0.955	0.478	0.812

Abbreviations/symbols. ^1^ Leaf gas exchange: Pn = net photosynthesis (μmol m^−2^ s^−1^); Ci = intercellular CO_2_ concentration (μmol mol^−1^); E = transpiration rate (mmol m^−2^ s^−1^); gs = stomatal conductance (mol m^−2^ s^−1^); J_CO_2__ = net photosynthesis expressed as electron transport rate (μmol m^−2^ s^−1^). ^2^ Fluorescence: efCO_2_ = electron use efficiency of photosynthesis (dimensionless); *Fv’/Fm’* = maximum efficiency of photosystem II in the light (dimensionless); Φ_PSII_ = effective quantum yield of photosystem II (dimensionless); ETR = electron transport rate (μmol m^−2^ s^−1^); J_NC_ = residual absorbed energy used for non-carboxylative processes (μmol m^−2^ s^−1^); efNC = electron use efficiency of non-carboxylative processes (dimensionless). ^3^ SPAD = indicator for chlorophyll content (SPAD units).

**Table 2:** Estimated marginal means (emmeans) of rootstock and crop load interactions (n = 3) for leaf gas exchange and chlorophyll fluorescence parameters of ‘Honeycrisp’ under Quincy (WA) growing conditions at 115 DAFB (season 2020). Emmeans followed by different letters were significantly different at *p* < 0.050 according to Tukey’s HSD test.

DAFB	Experimental Factor	Gas Exchange Variable ^1^		Fluorescence Variable ^2^		
		Pn	E	J_CO_2__	efCO_2_	efNC
115	R × C					
	M.9-T337 × Low	6.4 cd	1.34 ab	25.7 cd	0.316 ab	0.684 ab
	G.41 × Low	6.4 d	1.31 b	25.7 d	0.259 ab	0.741 ab
	G.935 × Low	7.2 bcd	0.93 b	28.9 bcd	0.438 a	0.699 ab
	M.9-T337 × High	13.0 cd	3.73 ab	51.9 cd	0.273 b	0.727 a
	G.41 × High	13.9 b	3.69 ab	55.7 b	0.307 ab	0.693 ab
	G.935 × High	18.5 a	4.76 a	73.8 a	0.301 ab	0.562 b
	*p* < 0.050	0.003	0.036	0.002	0.003	0.003

Abbreviations/symbols. ^1^ Leaf gas exchange: Pn = net photosynthesis (μmol m^−2^ s^−1^); E = transpiration rate (mmol m^−2^ s^−1^). ^2^ Fluorescence: J_CO_2__ = net photosynthesis expressed as electron transport rate (μmol m^−2^ s^−1^); efCO_2_ = electron use efficiency of photosynthesis (dimensionless); efNC = electron use efficiency of non-carboxylative processes (dimensionless).

## Data Availability

All data are contained within the article or available online as Supplementary Materials (Tables S1–S3).

## Supplementary Materials

The following supporting information can be downloaded at https://www.mdpi.com/article/10.3390/plants12234035/s1, Table S1. Estimated marginal means (emmeans) of ‘Honeycrisp’ tree performance parameters (average trunk cross sectional area (TCSA), number of fruit per tree at harvest, real crop load at harvest, net yield per tree, average fruit weight and yield efficiency) for rootstocks ‘G.41’, ‘G.935’, ‘M.9-T337’ (n = 6), low and high crop load levels (n = 9), and interactions between rootstock and crop load treatments (n = 3), under Quincy (WA) growing conditions in season 2020. Emmeans followed by different letters were significantly different at *p* < 0.050 according to Tukey’s HSD test.; Table S2: Means and standard deviations of agrometeorological parameters (wind speed, air temperature, solar radiation, and air relative humidity) recorded using an ATMOS 14 weather station located within the experimental rows, and equipped with an EM50 datalogger (Meter Group, Pullman, WA, USA) during gas exchange and chlorophyll fluorescence measurements at each date. Means and standard deviations were obtained using the values recorded by the weather station in the time window the measurements were collected (approximately between 10 a.m. and 1 p.m.); Table S3: Settings and readings of the infrared gas analyzer—LI-6400XT (LI-COR, Lincoln, NE, USA) equipped with a 2 cm^2^ leaf chamber with a LED light source—for gas exchange and chlorophyll fluorescence measurements at each date.
