# Supplementary material for: Rootstock and Crop Load Effects on ‘Honeycrisp’ Photosynthetic Performance and Carbohydrate Accumulation
Source: Plants (Basel). 2023 Nov 30;12(23):4035. doi: 10.3390/plants12234035 (PMC10708506; doi:10.3390/plants12234035)
Supplement: Supplementary file 1 [file plants-12-04035-s001.zip › plants-2664397-supplementary.pdf]

Table S1. Estimated marginal means (emmeans) of 'Honeycrisp' tree performance parameters (average trunk cross sectional area (TCSA), number of fruit per tree at harvest, real crop load at harvest, net yield per tree, average fruit weight and yield efficiency) for rootstocks 'G.41', 'G.935', 'M.9-T337' (n = 6), low and high crop load levels (n = 9), and interactions between rootstock and crop load treatments (n = 3), under Quincy (WA) growing conditions in season 2020. Emmeans followed by different letters were significantly different at p < 0.050 according to Tukey's HSD test. .Absence of letters for mean separation indicates non-significant differences.

Experimental factor	TCSA (cm^2)	No. fruit $tree^{-1}$	No. fruit cm^{-2} TCSA	Net yield (kg $tree^{-1}$)	Average fruit weight (g)	Yield efficiency (kg cm^{-2})
Rootstock (R)						
M.9-T337	12.8 b	65 c	5.8 b	15.1	234 a	1.18 a
G.41	14.7 a	92 a	6.9 a	14.2	152 b	0.96 b
G.935	14.3 a	78 b	6.4 ab	13.0	182 b	0.91 b
p < 0.050	< 0.001	< 0.001	0.048	0.086	< 0.001	< 0.001
Crop load (C)						
Low	13.9	46 b	3.3 b	10.8 b	242 a	0.78 b
High	13.9	131 a	9.4 a	17.4 a	136 b	1.26 a
p < 0.050	0.948	< 0.001	< 0.001	< 0.001	< 0.001	< 0.001
R × C						
M.9-T337 × Low	12.9 cd	37 d	2.9 b	11.0 cd	296 a	0.85 cd
G.41 × Low	14.4 bcd	57 c	4.0 b	10.2 d	180 b	0.71 d
G.935 × Low	14.6 bc	45 d	3.1 b	11.2 cd	251 a	0.77 d
M.9-T337 × High	12.8 d	112 b	8.8 a	19.2 a	172 bc	1.50 a
G.41 × High	15.0 b	147 a	9.9 a	18.2 ab	124 cd	1.21b
G.935 × High	14.0 bcd	134 a	9.6 a	14.8 bc	114 d	1.06 bc
p < 0.050	0.369	0.173	0.692	0.023	0.007	0.026

To assess the significance of tree performance parameters, a linear model was fitted on continuous type of data (TCSA, crop load at harvest, net yield, average fruit weight, and yield efficiency) with the lm:stats, and a generalized linear model was fitted on count data (number of fruit per tree at harvest), using nlme:glm [70]. In these models, rootstock and crop load level were treated as fixed effects. A two-way analysis of variance (ANOVA) with the type I sums of squares was conducted with R 'aov' base function to assess the effect of rootstock and crop load level on tree performance parameters. Means that were significantly different were separated by Tukey's HSD (α = 0.05), using emmeans:emmeans [72]. All means reported, also obtained with the 'emmeans' function, are estimated marginal means.

Table S2. Means and standard deviations of agrometeorological parameters (wind speed, air temperature, solar radiation, air relative humidity) recorded by an ATMOS 14 weather station located within the experimental rows, and equipped with an EM50 datalogger (Meter Group, Pullman, WA, USA) during gas exchange and chlorophyll fluorescence measurements at each date. Means and standard deviations were obtained using the values recorded by the weather station in the time window the measurements were collected (approximately between 10 a.m. and 1 p.m.).

Date	DAFB	Wind speed ($m\ s^{-1}$)	Air temperature (°C)	Solar radiation ($W\ m^{-2}$)	Air relative humidity (%)
25/06/2020	65	0.04 ± 0.05	30.4 ± 2.5	1188 ± 275	37.9 ± 5.9
30/06/2020	70	0.31 ± 0.18	26.4 ± 1.9	1210 ± 272	35.9 ± 4.3
13/07/2020	83	0.03 ± 0.05	23.8 ± 1.4	1207 ± 261	36.7 ± 4.1
28/07/2020	98	0.02 ± 0.04	34.8 ± 2.9	1121 ± 193	33.7 ± 7.3
14/08/2020	115	0.03 ± 0.04	26.2 ± 1.9	1118 ± 192	37.9 ± 2.8
28/08/2020	129	0.04 ± 0.05	27.4 ± 2.1	880 ± 321	37.1 ± 5.0
30/09/2020	162	0.03 ± 0.05	24.8 ± 3.5	657 ± 185	45.2 ± 9.1

Table S3. Settings and readings of the infrared gas analyzer – LI-6400XT (LI-COR, Lincoln, NE, USA) equipped with a 2 cm^2 leaf chamber with a LED light source – for gas exchange and chlorophyll fluorescence measurements at each date.

Date	DAFB	Photosynthetic Photon Flux Density (PPFD $\mu mol\ m^{-2}\ s^{-1}$)	Temperature (°C)	Reference CO_2 ($\mu mol\ CO_2\ mol^{-1}$)	Flow rate ($\mu mol\ s^{-1}$)
25/06/2020	65	2050	31.0	400	400
30/06/2020	70	2100	27.0	400	400
13/07/2020	83	1835	25.0	400	400
28/07/2020	98	1640	35.0	400	400
14/08/2020	115	1230	24.5	400	400
28/08/2020	129	1770	26.0	400	400
30/09/2020	162	1400	23.5	400	400
